# Supplementary material for: DNase II mediates a parthanatos-like developmental cell death pathway in Drosophila primordial germ cells
Source: Nat Commun. 2021 Apr 16;12:2285. doi: 10.1038/s41467-021-22622-1 (PMC8052343; doi:10.1038/s41467-021-22622-1)
Supplement: Supplementary file 1 — Supplementary Information [file 41467_2021_22622_MOESM1_ESM.pdf]

DNase II mediates a parthanatos-like developmental cell death pathway in *Drosophila* primordial germ cells

Tarayrah-Ibraheim et al.

Supplementary Information

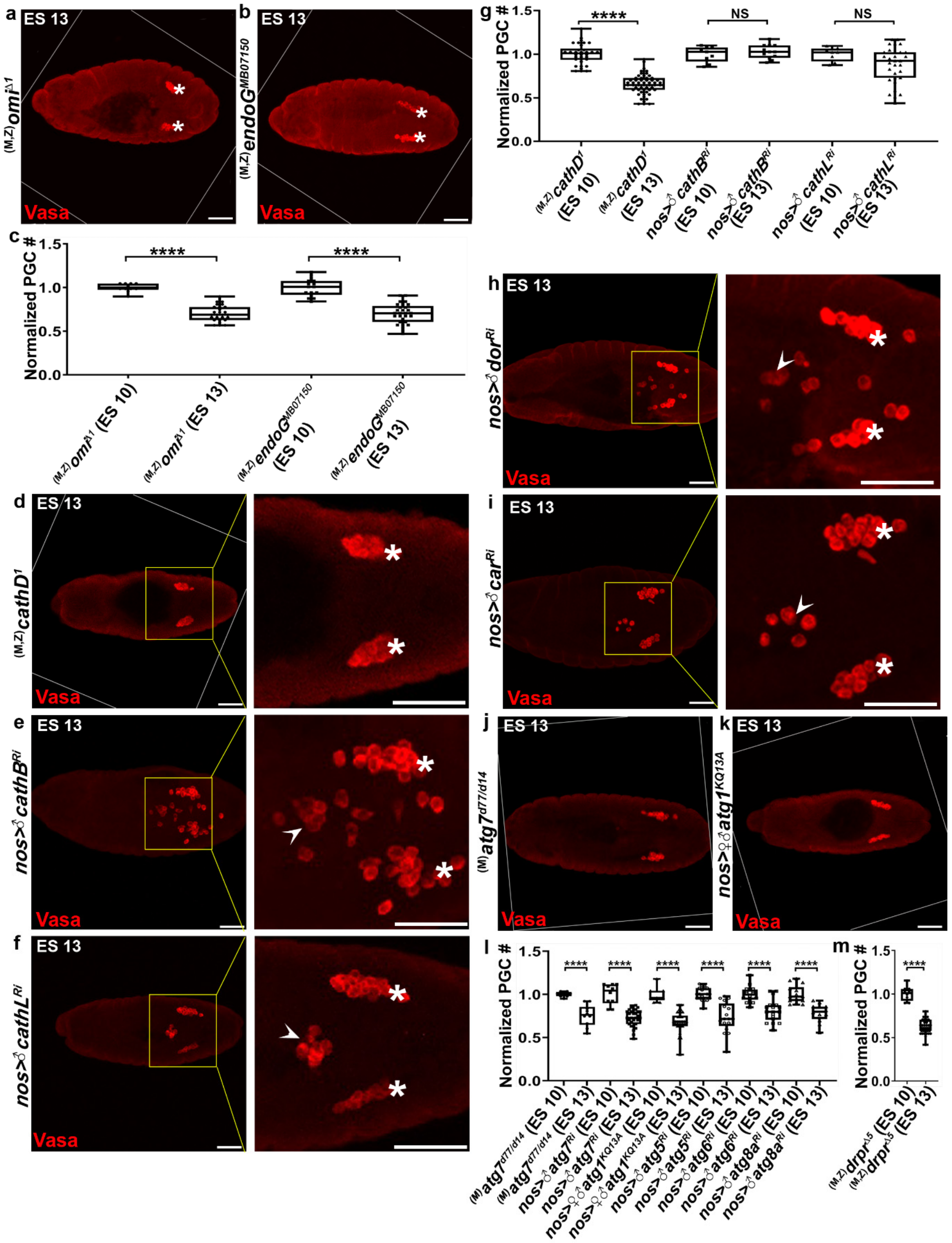

Supplementary Fig. 1 PGC death is distinct from germ cell death (GCD) and autophagy-dependent cell death (ADCD). a-c, Inactivation of two mitochondrial enzymes which mediate GCD, Omi and EndoG, had no effect on PGC death. Shown are representative images of ES 13 embryos mutant for *omi* (**a**) and *endoG* (**b**) stained to visualize the PGCs (Vasa; red). Asterisks

indicate gonadal PGCs. Note the absence of ectopically surviving PGCs at the embryonic midline region. Scale bars, 50 µm. **c**, The corresponding quantifications of the PGC death levels. All data points, including outliers, were presented in box plot format where the minimum is the lowest data point represented by the lower whisker bound, the maximum is the highest data point represented by the upper whisker bound, and the center is the median. The lower box bound is the median of the lower half of the dataset while the upper box bound is the median of the upper half of the dataset. Each dot corresponds to the number of PGCs in a single embryo to reflect n number, where n=number of examined biologically independent embryos. ****$p < 0.0001$; Student's t-test, one-sided distribution. **d-g**, The lysosomal cathepsins B and L, but not D, are involved in PGC death. Shown are representative images of ES 13 embryos either mutant for *cathD* (**d**) or with PGC-specific knockdowns of *cathB* (**e**) or *cathL* (**f**), stained to visualize the PGCs (Vasa; red) and presented as in Fig. 1b. Arrowheads pointing at ectopically surviving midline PGCs. Asterisks indicate gonadal PGCs. Scale bars, 50 µm. Note that in contrast to its major effect on GCD[1], the *cathD* mutant had no effect on PGC death. The corresponding quantifications of the PGC death levels (**g**) were calculated and presented as in (**c**). ****$p < 0.0001$; NS, non-significant; Student's t-test, one-sided distribution. **h**,**i**, PGC lysosomal biogenesis is required for PGC death. Shown are ES 13 embryos with PGC-specific knockdowns of *dor* (**h**) and *car* (**i**), displaying strong PGC death attenuation (arrowheads pointing at ectopically surviving midline PGCs). Asterisks indicate gonadal PGCs. Scale bars, 50 µm. The corresponding quantifications are presented in Fig. 2k. **j-l**, Inactivation of major autophagy pathway genes functioning in the initiation complex (Atg1), nucleation complex (Atg6), and elongation complexes (Atg5, 7, and 8), all had no significant effect on PGC death levels. Shown are representative images of ES 13 embryos mutant for *atg7* (**j**) or with PGC-specific expression of a dominant-negative form of Atg1 (**k**), stained, annotated and presented as in (**a**). Note the absence of ectopically surviving PGCs at the embryonic midline region. Scale bars, 50 µm. The quantifications of PGC death levels in autophagy compromised embryos and PGCs (**l**) were calculated and presented as in (**c**). ****$p < 0.0001$; Student's t-test, one-sided distribution. **m**, Quantification of PGC death levels in embryos lacking the major phagocytic receptor Drpr. Calculated and presented as in (**c**). ****$p < 0.0001$; Student's t-test, one-sided distribution.

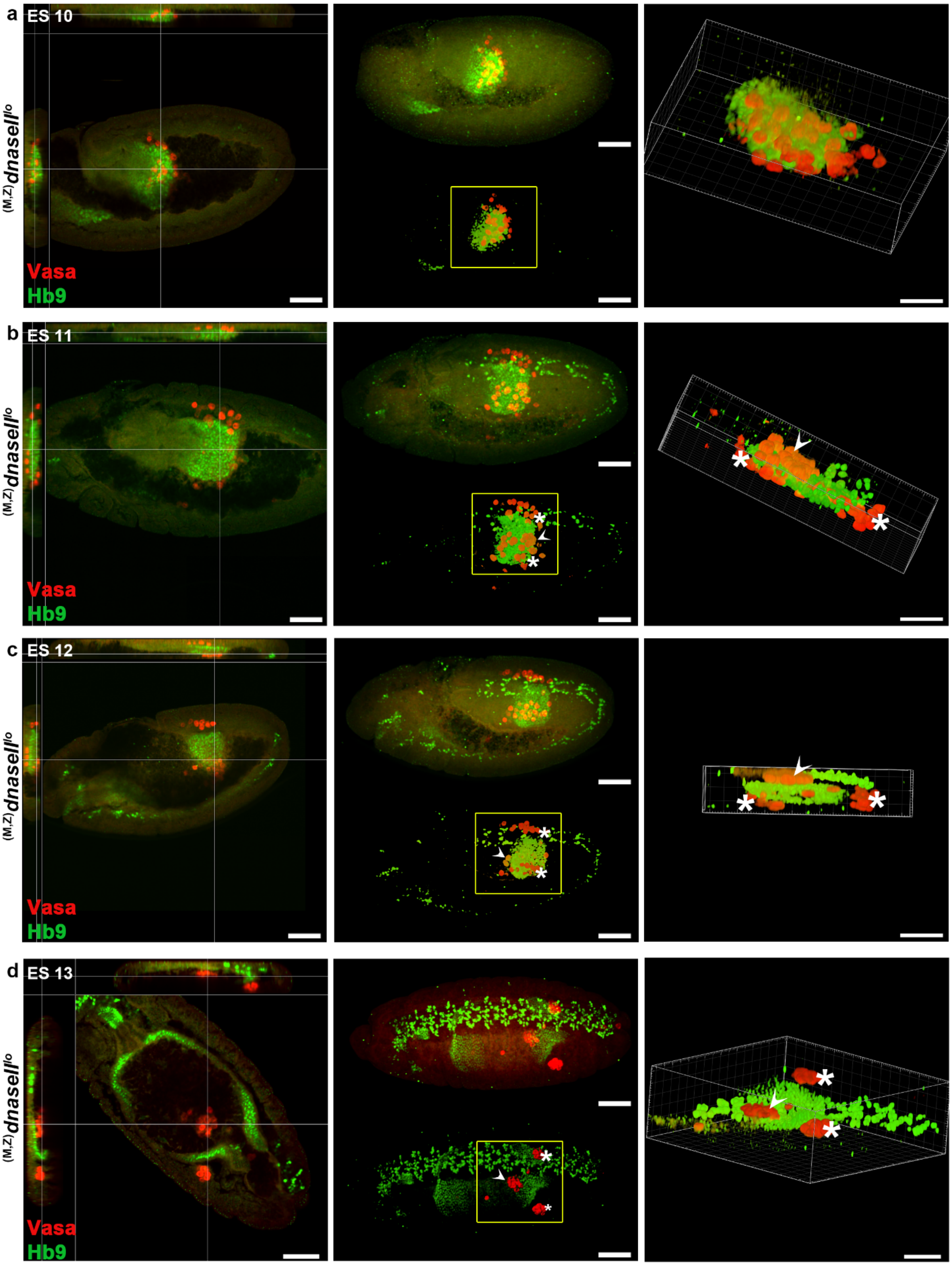

Supplementary Fig. 2 DNase II is not required for PGC migration. a-d, *dnaseII* mutant PGCs exit the midgut. Representative images of *dnaseII* mutant embryos at ES 10-13, respectively,

stained to visualize the PGCs (Vasa; red) and the midgut (Hb9; green). Asterisks indicate gonadal PGCs; arrowheads indicate ectopically surviving PGCs. Scale bars, 30 μm. The representative embryos at each stage were shown as a cross-section (left panels), a maximum intensity projection (top middle panels) and its volume rendering (bottom middle panels), and higher magnification of the volume rendering rotated to obtain the best view (right panels).

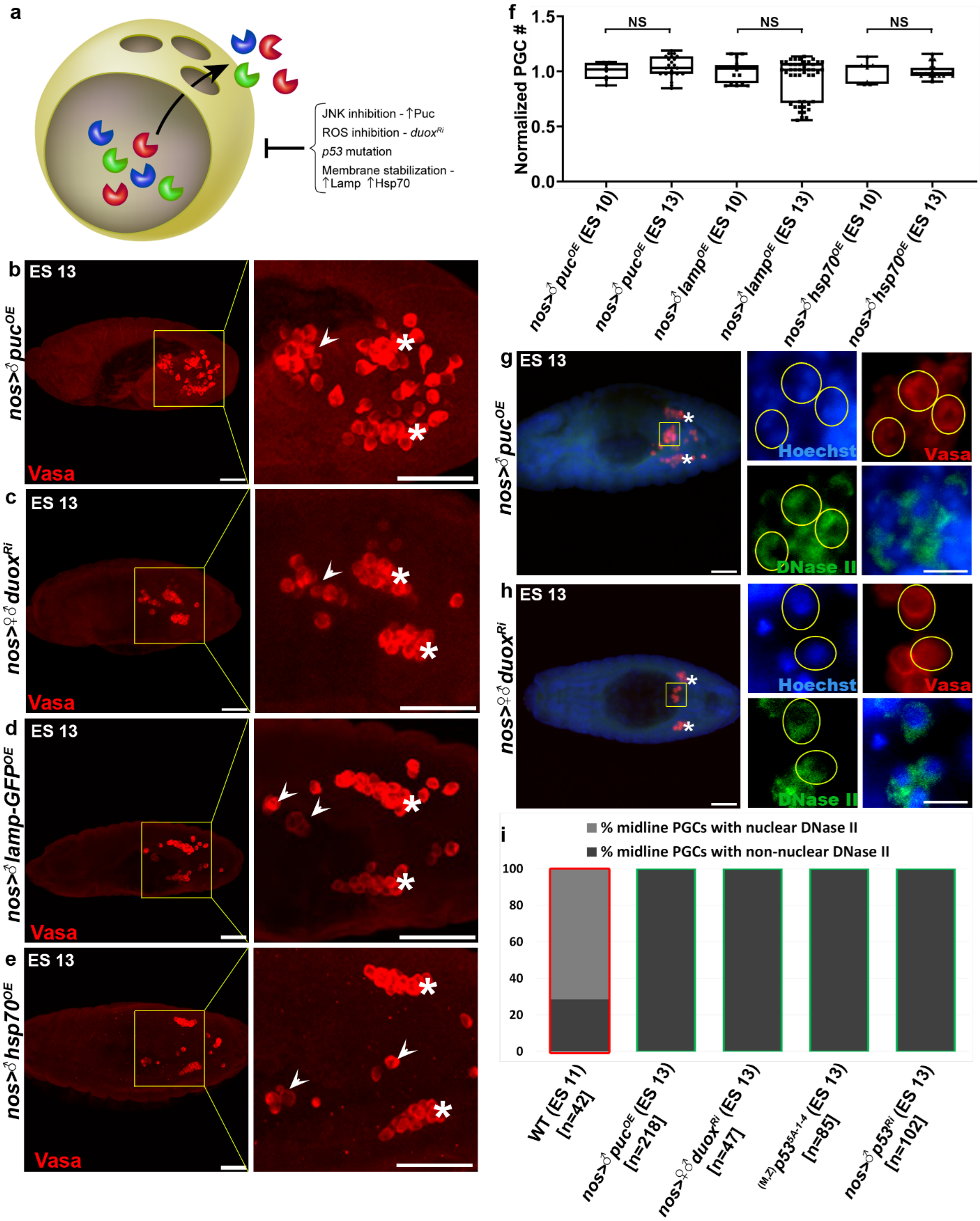

Supplementary Fig. 3 Functional LMP pathways are required for nuclear translocation of DNase II and PGC death. a, Illustration of a lysosome undergoing LMP, featuring the genetic manipulations performed to compromise PGC LMP. **b-f**, Inactivation of several genetic pathways involved in LMP attenuate PGC death. Representative images of ES 13 embryos with the genetic manipulations listed in (**a**), stained and presented as in Fig. 1b. PGCs (Vasa; red). Arrowheads

point at ectopically surviving midline PGCs. Asterisks indicate gonadal PGCs. Scale bars, 50 µm. Shown are ES 13 embryos genetically manipulated as follows: Inhibition of the Jun N-terminal kinase (JNK) signaling pathway through OE of Puckered (Puc), the sole JNK-specific MAPK phosphatase (MKP) in *Drosophila*[2] (**b**); Inhibition of reactive oxygen species (ROS) production, by knocking down a major ROS generating enzyme, Dual oxidase (Duox; **c**); Stabilizing the lysosomal membrane by PGC-specific OE of the GFP-fused Lysosome-associated membrane protein 1 (Lamp-1; **d**) or OE of the Heat shock protein 70 (Hsp70; **e**). Additional data concerning p53 inactivation are presented in Fig. 3h-m. **f**, Quantification of PGC death levels in embryos corresponding to the genotypes in (**b**,**d**,**e**). All data points, including outliers, were presented in box plot format where the minimum is the lowest data point represented by the lower whisker bound, the maximum is the highest data point represented by the upper whisker bound, and the center is the median. The lower box bound is the median of the lower half of the dataset while the upper box bound is the median of the upper half of the dataset. Each dot corresponds to the number of PGCs in a single embryo to reflect n number, where n=number of examined biologically independent embryos. NS, non-significant; Student's *t*-test, one-sided distribution. **g-i**, Compromised LMP block DNase II nuclear translocation in the ectopically surviving midline PGCs. Representative images of ES 13 embryos with PGC-specific *puc* OE (**g**) and *duox* knockdown (**h**), stained, annotated and presented as in Fig. 3k,l. Scale bars, 50 µm. Note the non-nuclear DNase II localization in the surviving midline PGCs. Scale bars, 10 µm. **i**, Quantification of the percentage of midline PGCs with nuclear versus non-nuclear DNase II localization for embryos of the indicated genotypes. Green column outlines indicate that the cells being counted are living PGCs, red column outline indicates that the cells being counted are dying PGCs. n number is shown in brackets where n=number of examined PGCs.

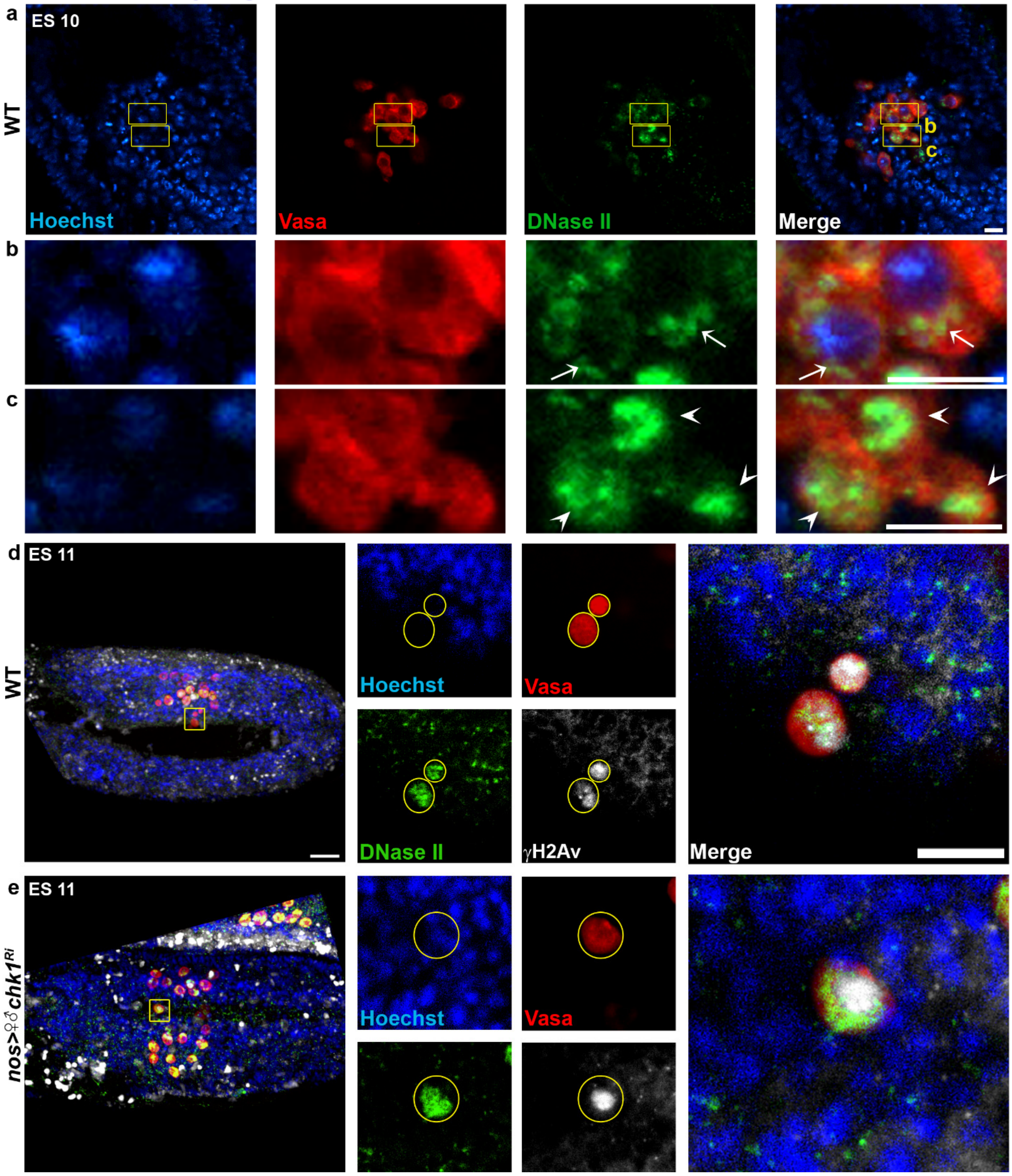

Supplementary Fig. 4 Nuclear translocation of DNase II is associated with DNA breaks and is partially dependent on the DDR pathway. a-c, DNase II translocates to the nucleus in dying PGCs. Shown are a midline region of a WT embryo, at ES 10, stained to visualize the DNA (Hoechst; blue), PGCs (Vasa; red) and DNase II (green) (**a**), and magnifications of the areas outlined by yellow rectangles in (**b**,**c**). Whereas in living PGCs, DNase II is confined to the lysosomes within the cytoplasmic compartment (arrows), strong DNase II expression is detected in the nuclei of dying PGCs (arrowheads). Scale bars, 10 µm. **d**,**e**, Translocation of DNase II to

the nucleus elicits DNA fragmentation. Shown are ES 11 WT (**d**) and PGC-specific *chk1* knockdown (**e**) embryos stained to visualize the PGCs (Vasa; red), γH2Av (white), DNase II (green), and DNA (Hoechst, blue). The outlined area (yellow square) is magnified in the middle panels and further on in the right panels. Scale bars, 50 µm. Midline PGCs are outlines (yellow circles). Note the nuclear area co-localization between DNase II and γH2Av in WT dying midline PGCs (**d**), as opposed to only partial nuclear localization of DNase II in the *chk1* knockdown ectopically surviving midline PGC (**e**). Scale bars, 10 µm.

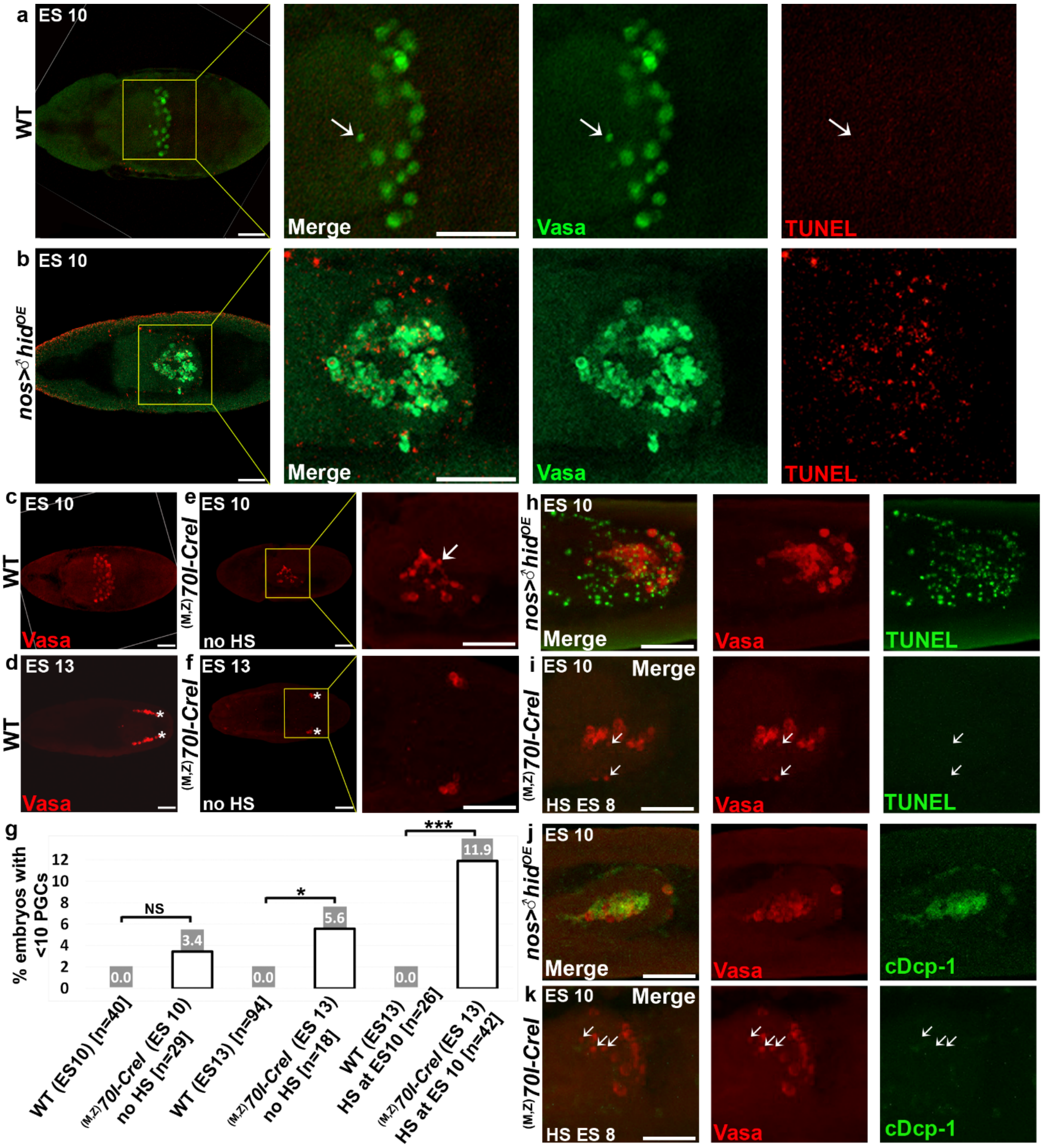

Supplementary Fig. 5 Developmentally dying midline PGCs and PGCs dying precociously following OE of a homing endonuclease are both refractory to TUNEL staining. a,b, Developmentally dying midline PGCs, but not *hid* overexpressing apoptotic PGCs, are TUNEL-negative. Representative images of ES 10 WT (**a**) and PGC-specific *hid* OE (**b**) embryos labeled with TUNEL (red). PGCs (Vasa; green). The outlined areas (yellow squares) are magnified in the right panels. The arrow is pointing at a TUNEL-negative highly condensed dying PGC. Scale bars, 50 µm. **c-k**, PGC-specific ectopic expression of the homing endonuclease I-*Cre*I induces precocious non-apoptotic PGC death. Shown are representative images of WT embryos at ES 10 (**c**) and ES 13 (**d**), and embryos carrying the *70I-CreI* transgene without heat shock treatment (no

HS), at ES 10 (**e**) and ES 13 (**f**), stained and presented as in Fig. 1b. PGCs (Vasa; red). Asterisks indicate gonadal PGCs. Note that in some of the embryos, even without HS, the leakiness of the transgene induced precocious PGC death visualized by the excessive number of dying PGCs at ES 10 (an arrow) and the very few PGCs that populate the gonads at ES 13. Scale bars, 50 µm. **g**, Quantification of the percentage of embryos with less than 10 PGCs in WT and in embryos carrying the *70I-CreI* transgene, with and without HS treatment. The percent value is indicated above each column. n number is shown in brackets where n=number of examined biologically independent embryos. ***p < 0.001; *p < 0.05; NS, non-significant; Fisher's test. **h-k**, Representative images of the midline regions of ES 10 embryos overexpressing *hid* (**h**,**j**) or embryos carrying the *70I-CreI* transgene and treated with HS at ES 8 (**i**,**k**), stained for TUNEL (green) (**h**,**i**) or for activated caspases (cleaved Dcp-1; cDcp-1) (**j**,**k**). PGCs (Vasa; red). Arrows pointing at TUNEL- and cDcp-1-negative dying PGCs. Scale bars, 50 µm.

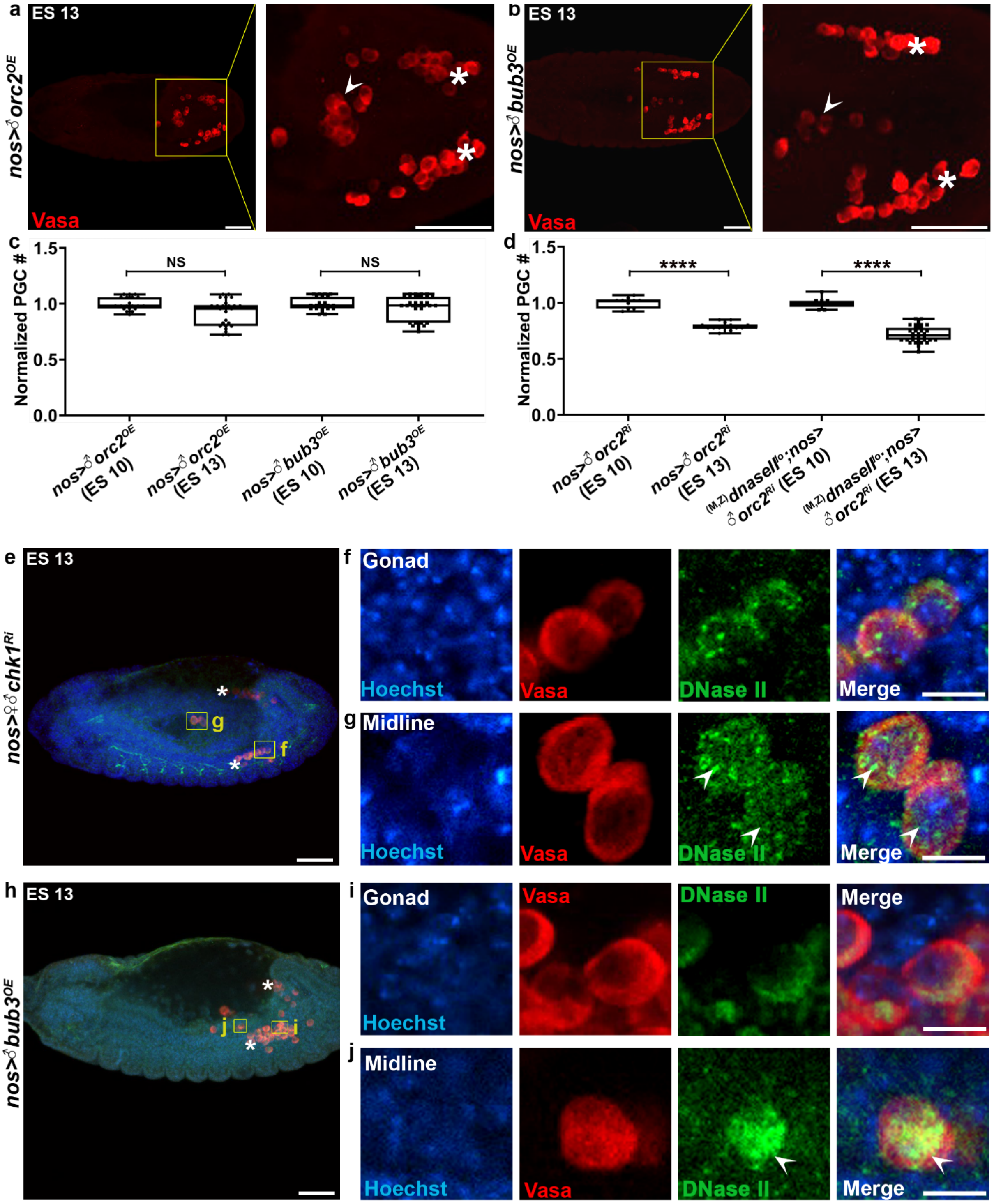

Supplementary Fig. 6 Checkpoint pathways are involved in PGC death in parallel and downstream of DNase II nuclear translocation. a-c, Manipulating the DNA replication and the spindle assembly checkpoints attenuate PGC death. Shown are representative images of embryos with PGC-specific OE of the DNA replication checkpoint component, Orc2 (**a**), and the spindle assembly checkpoint component, Bub3 (**b**), stained and presented as in Fig. 1b. Arrowheads pointing at ectopically surviving midline PGCs. Asterisks indicate gonadal PGCs. The

corresponding quantification of PGC death levels is shown in (**c**). All data points, including outliers, were presented in box plot format where the minimum is the lowest data point represented by the lower whisker bound, the maximum is the highest data point represented by the upper whisker bound, and the center is the median. The lower box bound is the median of the lower half of the dataset while the upper box bound is the median of the upper half of the dataset. Each dot corresponds to the number of PGCs in a single embryo to reflect n number, where n=number of examined biologically independent embryos. NS, non-significant; Student's *t*-test, one-sided distribution. **d**, Manipulating the DNA replication checkpoint restores normal levels of PGC death in *dnaseII* mutant embryos. Quantification of PGC death levels in WT and *dnaseII* mutant embryos following PGC-specific knockdown of *orc2* calculated and presented as in (**c**). ****$p < 0.0001$; Student's *t*-test, one-sided distribution. Note that whereas knockdown of *orc2* restores PGC death in the *dnaseII* mutant embryos to about normal levels, it does not cause ectopic PGC death on its own. **e-j**, DNase II translocates to the nucleus of ectopically surviving midline PGCs following DDR and spindle assembly checkpoints manipulations. Shown are representative images of embryos with PGC-specific *chk1* knockdown (**e**) or OE of *bub3* (**h**), stained to visualize the DNA (Hoechst; blue), PGCs (Vasa; red) and DNase II (green), and magnifications of corresponding areas outlined by yellow rectangles (**f**,**g**,**i**,**j**). Asterisks indicate gonadal PGCs. Arrowheads pointing at nuclear DNase II. Note that whereas nuclear translocation of DNase II was almost complete upon spindle assembly checkpoint manipulation (**j**), this translocation was only partial when the DDR checkpoint was compromised (**g**), suggesting that the spindle assembly checkpoint acts downstream of DNase II nuclear translocation, while the DDR pathway is required for the massive nuclear translocation of DNase II. Scale bars in (**e**,**h**), 50 µm; (**f**,**g**,**i**,**j**), 10 µm.

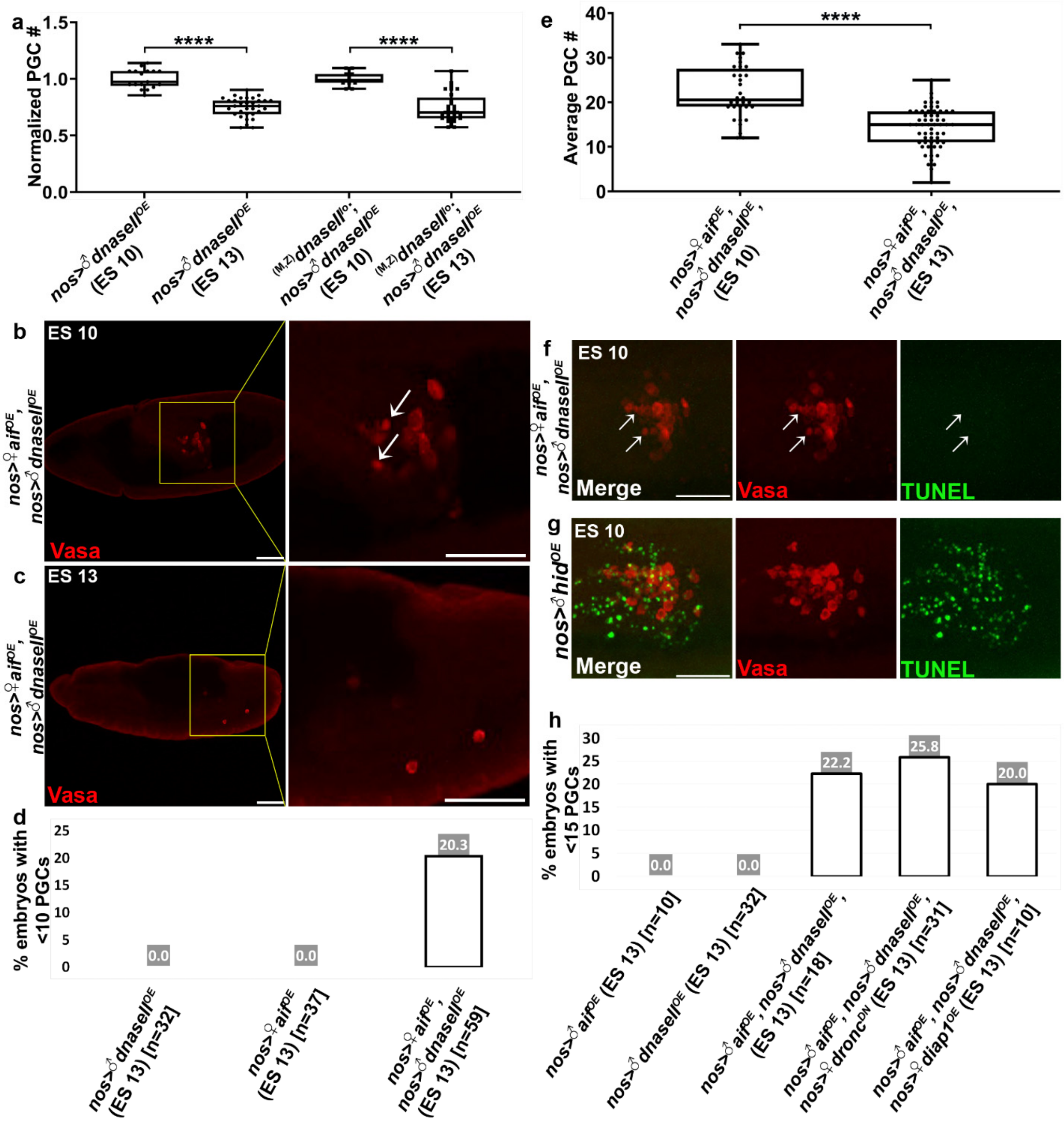

Supplementary Fig. 7 AIF and DNase II cooperatively trigger non-apoptotic PGC death. a, OE of *dnaseII* opposed the block in PGC death caused by the *dnaseII*^{lo} mutation, confirming the validity of the *dnaseII* OE transgene. Shown is quantification of PGC death levels in WT (left) and *dnaseII* mutant (right) embryos following PGC-specific OE of *dnaseII*. All data points, including outliers, were presented in box plot format where the minimum is the lowest data point represented by the lower whisker bound, the maximum is the highest data point represented by the upper whisker bound, and the center is the median. The lower box bound is the median of the lower half of the dataset while the upper box bound is the median of the upper half of the dataset. Each dot corresponds to the number of PGCs in a single embryo to reflect n number, where n=number of examined biologically independent embryos. ****p < 0.0001; Student's t-test, one-sided distribution. **b**,**c**, PGC-specific double OE of *aif* and *dnaseII* induces precocious cell death. Shown

are representative images of ES 10 (**b**) and ES 13 (**c**) embryos with PGC-specific double OE of *aif* and *dnaseII*, stained and presented as in Fig. 1b. Arrows pointing at highly condensed dying PGCs detected already at ES 10. Note the significant reduction in the overall number of PGCs at ES 13. Scale bars, 50 µm. **d**, Quantification of the percentage of ES 13 embryos with less than 10 PGCs following PGC-specific OE of *aif*, *dnaseII* or both. The percent value is indicated above each column. n number is shown in brackets where n=number of examined biologically independent embryos. **e**, Developmental cell death of one third of the PGCs is maintained even when the initial PGC pool at ES 10 is low due to precocious PGC death. Quantification of the average number of PGCs in ES 10 and 13 embryos following PGC-specific double OE of *aif* and *dnaseII*. The average PGC numbers were calculated and presented in box plot format as in (**a**). Each dot corresponds to the average number of PGCs in a single embryo to reflect n number, where n= number of examined biologically independent embryos. ****$p < 0.0001$; Student's *t*-test, one-sided distribution. **f**,**g**, Double OE of *aif* and *dnaseII* triggers non-apoptotic PGC death. Shown are representative images of the midline regions of ES 10 embryos following either PGC-specific double OE of *aif* and *dnaseII* (**f**) or *hid* (**g**), labeled with TUNEL (green). PGCs (Vasa; red). Arrows pointing at TUNEL-negative dying PGCs. Note the multiple TUNEL-positive dying PGCs following *hid* OE. Scale bars, 50 µm. **h**, Precocious PGC death induced by double OE of *aif* and *dnaseII* is not attenuated by OE of potent caspase inhibitory proteins. Quantification of the percentage of ES 13 embryos with less than 15 PGCs following PGC-specific OE of *aif*, *dnaseII* or both, with or without OE of the caspase inhibitory proteins, Diap1 and Dronc^{DN}. The percent value is indicated above each column. n number is shown in brackets where n=number of examined biologically independent embryos.

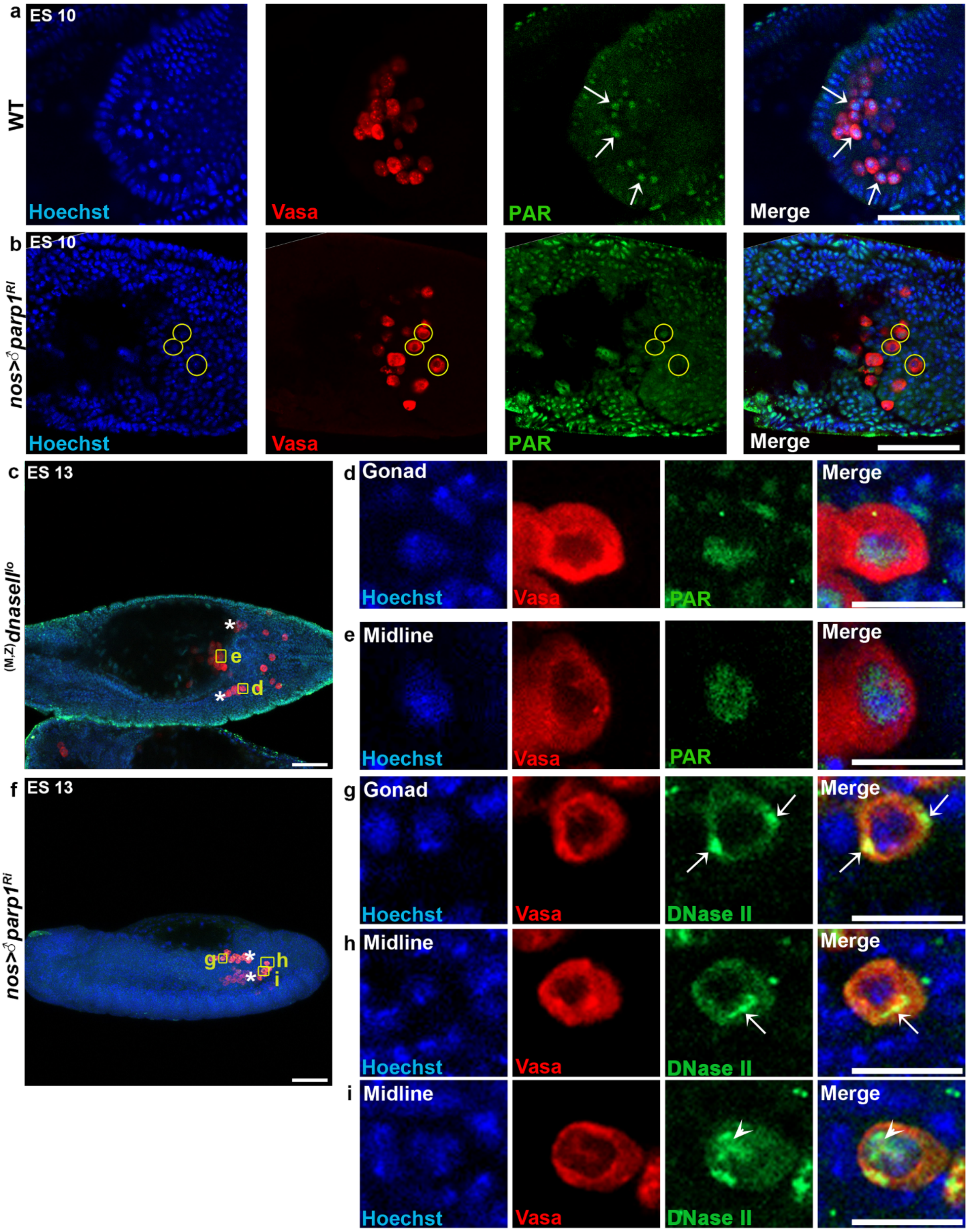

Supplementary Fig. 8 PARP-1 and DNase II mutually affect each other's activity. a,b, Accumulation of PAR polymers in the PGCs requires PARP-1 activity. Representative images of the midline regions of a WT (**a**) and a PGC-specific *parp1* knockdown (**b**) embryos at ES 10 stained to visualize PAR (anti-PAR; green), PGCs (Vasa; red), and DNA (Hoechst; blue). Arrows

pointing at WT PGCs displaying strong nuclear PAR signal. Circled, are three *parp1* knockdown PGCs, two of which have no PAR, whereas the third displays a highly reduced PAR signal. Scale bars, 50 μm. **c-e**, PARP-1 overactivation and the release of PAR to the cytoplasm require functional DNase II. Shown are a representative image of an ES 13 *dnaseII* mutant embryo (**c**), stained as in (**a**), and magnifications of the areas outlined by yellow rectangles, featuring a gonadal PGC (**d**) and an ectopically surviving midline PGC (**e**). Asterisks indicate gonadal PGCs. Note that the release of PAR from the nucleus of midline PGCs is completely blocked in these mutants. Scale bars in (**c**), 50 μm; (**d**,**e**), 10 μm. **f-i**, DNase II nuclear translocation requires functional PARP-1. Shown are a representative image of an ES 13 embryo with PGC-specific *parp1* knockdown (**f**), stained to visualize DNase II (green), PGCs (Vasa; red), and DNA (Hoechst; blue), and magnifications of the areas outlined by yellow rectangles, featuring a gonadal PGC (**g**) and two ectopically surviving midline PGCs (**h**,**i**). Asterisks indicate gonadal PGCs. Arrows pointing at cytoplasmic DNase II, while the arrowhead points at nuclear DNase II. Scale bars in (**f**), 50 μm; (**g-i**), 10 μm.

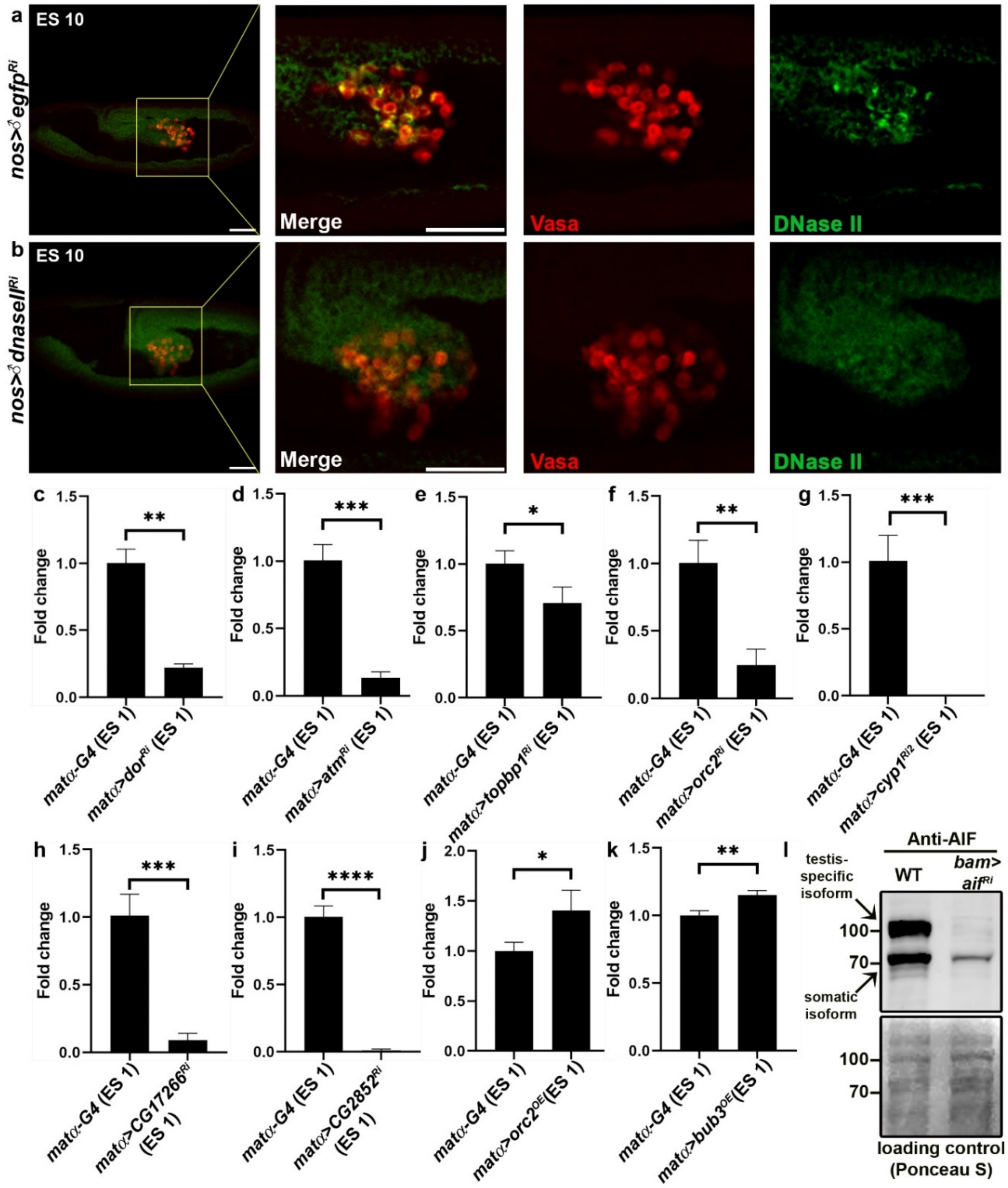

Supplementary Fig. 9 Validation of the knockdown and overexpression lines used in this study. a,**b**, Representative images of ES 10 embryos with PGC-specific knockdowns of *egfp* (**a**) and *dnaseII* (**b**) stained to visualize the PGCs (Vasa; red) and DNase II (anti-DNase II antibodies; green). The outlined areas (yellow squares) are magnified in the right panels. Scale bars, 50 µm. **c-k**, Quantitative RT-PCR analysis in embryos with maternal knockdown of *dor* (**c**), *atm* (**d**), *topbp1* (**e**), *orc2* (**f**), *cyp1* (**g**), *CG17266* (**h**), and *CG2852* (**i**), and maternal overexpression of *orc2* (**j**) and *bub3* (**k**). ****$p < 0.0001$; $0.0001 <$ ***$p < 0.001$; $0.001 <$ **$p < 0.01$; $0.01 <$ *$p < 0.1$; Two tailed unpaired student's *t*-test. (**l**) Western blot analysis of AIF protein levels in WT testes compared to testes with germ cell specific *aif* knockdown. Ponceau S stain was used for loading control.

Supplementary Discussion

The specific molecular mechanism by which the DDR mediates cell death in this instance is unknown. Chk1 is a serine-threonine protein kinase, which is selectively phosphorylated and activated by ATR to trigger a wide range of distinct downstream responses, including transcription regulation, energy consumption alteration, cell cycle arrest/delay, DNA repair and cell death[3–5]. Interestingly, dozens of Chk1 substrates, as well as numerous Chk1 interacting proteins have been reported[5]. Furthermore, a large-scale proteomic analysis identified hundreds of ATM and ATR potential substrates[6]. It is therefore reasonable to propose that some of these targeted proteins could be directly involved in the execution of PGC death.

PGCs have been the subject of many studies during the past three decades, mainly because of their profound migration capacity[7]. Several genes involved in PGC biology have been identified, of which four *Drosophila* genes, *wunen* (*wun*) and *wun2*, *outsiders* (*out*), and *p53*, also affected PGC death[8–12], although the underlying cell death mechanisms remained elusive. The notion that PGCs undergo cell death by parthanatos now allows for renewed speculation regarding the possible functions of these genes. Indeed, the discovery that genetic LMP pathways mediate PGC death now provides an explanation for the role of p53, highlighting its less explored capacity to promote LMP, rather than its function as an effector of the DDR. Furthermore, since PARP-1 generates PAR polymers by catalyzing the polymerization of ADP-ribose units from donor nicotinamide adenine dinucleotide (NAD^+) molecules, it is possible that mutations in the *out* gene, which encodes a putative monocarboxylate transporter (MCT), attenuate PGC death by significantly decreasing the cellular pool of NAD^+. This hypothesis is based on a recent report, showing that inhibition of at least some MCTs in cancer cells leads to accumulation of high intracellular lactate, which in turn inhibits lactate dehydrogenase, one of two enzymes regenerating NAD^+ from NADH[13]. Finally, in contrast to p53 and Out that affect PGC death but not migration, the lipid phosphate phosphatases Wun and Wun2, appear to act further upstream, regulating both lateral PGC migration and cell death of the PGCs that fail to migrate and remain in the midline.

Although apoptosis is the main form of cell death during development, emerging but still scarce evidence suggests that other forms of cell death are preferentially utilized by certain developmental systems despite their ability to trigger apoptosis[1,14–20]. Why some cells die by an ACD pathway even though they are capable of activating caspases and undergoing apoptosis is an important, unresolved question. One explanation could be that some forms of ACD are more beneficial to the organism than others, as is thought to be the case with pyroptosis, which is a proinflammatory and lytic mode of cell death, believed to emerge as a key defense against microbial infections[21,22]. However, for most of the known paradigms, the advantages of ACDs over apoptosis during development are far less obvious. For example, at least superficially, parthanatos and apoptosis share many anatomical and morphological features, including significant cell shrinkage, nuclear condensation and DNA fragmentation. These imply that cell death by parthanatos might hold at least some unknown advantages over apoptosis, or that being highly sensitive to apoptosis might be a drawback for some cells. Consistent with the latter idea, it is now well established that certain cells utilize apoptotic caspases in a variety of non-lethal cellular processes (generally dubbed CDPs), and thus require tight control over the apoptotic machinery to prevent excessive caspase activity and unwanted cell death[23–25]. Along this line, recent work from our lab uncovered an apoptosis-independent role of caspases as potent inhibitors of cell migration and invasion, where

even mild elevation of basal caspase activity levels in the PGCs significantly inhibited PGC migration[26]. Since PGCs must migrate across the embryo to reach the somatic gonadal precursors and form the testes and ovaries[7], it is attractive to propose that PGCs might avoid apoptosis to minimize the risk of caspases interfering with cell migration. Consistent with this idea, the linker cell in *C. elegans*, which also activates a *bona-fide* caspase-independent cell death program during development, follows a stereotypical migration path before dying, as it migrates from where it is born to a position near the sperm exit channel. It would be highly interesting to examine possible non-apoptotic roles of caspases during linker cell development[14,19,20]. Given that dozens of CDPs have been identified and characterized in a variety of cell types and organisms[23,27], the idea that CDPs and ACDs might be two sides of the same coin could therefore allow for more targeted explorations and possible discoveries of new developmental paradigms involving ACD pathways.

Supplementary References

1. Yacobi-Sharon, K., Namdar, Y. & Arama, E. Alternative germ cell death pathway in drosophila involves HtrA2/Omi, lysosomes, and a caspase-9 counterpart. *Dev. Cell* **25**, 29–42 (2013).
2. Martin-Blanco, E. *et al.* puckered encodes a phosphatase that mediates a feedback loop regulating JNK activity during dorsal closure in Drosophila. *Genes Dev.* **12**, 557–570 (1998).
3. Smith, J., Tho, L. M., Xu, N. & Gillespie, D. A. The ATM-Chk2 and ATR-Chk1 pathways in DNA damage signaling and cancer. *Adv.Cancer Res.* **108**, 73–112 (2010).
4. Bartek, J. & Lukas, J. Chk1 and Chk2 kinases in checkpoint control and cancer. *Cancer Cell* **3**, 421–429 (2003).
5. Zhang, Y. & Hunter, T. Roles of Chk1 in cell biology and cancer therapy. *Int. J. Cancer* **134**, 1013–1023 (2014).
6. Matsuoka, S. *et al.* ATM and ATR substrate analysis reveals extensive protein networks responsive to DNA damage. *Science* **316**, 1160–1166 (2007).
7. Richardson, B. E. & Lehmann, R. Mechanisms guiding primordial germ cell migration: strategies from different organisms. *Nat.Rev.Mol.Cell Biol.* **11**, 37–49 (2010).
8. Sano, H., Renault, A. D. & Lehmann, R. Control of lateral migration and germ cell elimination by the Drosophila melanogaster lipid phosphate phosphatases Wunen and Wunen 2. *J. Cell Biol.* **171**, 675–683 (2005).
9. Yamada, Y., Davis, K. D. & Coffman, C. R. Programmed cell death of primordial germ cells in Drosophila is regulated by p53 and the Outsiders monocarboxylate transporter. *Development* **135**, 207–216 (2008).
10. Renault, A. D., Sigal, Y. J., Morris, A. J. & Lehmann, R. Soma-germ line competition for lipid phosphate uptake regulates germ cell migration and survival. *Science (80-.).* **305**, 1963–1966 (2004).
11. Hanyu-Nakamura, K., Kobayashi, S. & Nakamura, A. Germ cell-autonomous Wunen2 is required for germline development in Drosophila embryos. *Development* **131**, 4545–4553 (2004).
12. Renault, A. D., Kunwar, P. S. & Lehmann, R. Lipid phosphate phosphatase activity regulates dispersal and bilateral sorting of embryonic germ cells in Drosophila. *Development* **137**, 1815–1823 (2010).
13. Benjamin, D. *et al.* Dual Inhibition of the Lactate Transporters MCT1 and MCT4 Is Synthetic Lethal with Metformin due to NAD+ Depletion in Cancer Cells. *Cell Rep.* **25**, 3047-3058.e4 (2018).
14. Blum, E. S., Abraham, M. C., Yoshimura, S., Lu, Y. & Shaham, S. Control of nonapoptotic developmental cell death in Caenorhabditis elegans by a polyglutamine-repeat protein. *Science (80-.).* **335**, 970–973 (2012).
15. Denton, D. *et al.* Autophagy, not apoptosis, is essential for midgut cell death in Drosophila. *Curr.Biol* **19**, 1741–1746 (2009).

16. Sahu, R. *et al.* Drosophila embryos as model systems for monitoring bacterial infection in real time. *Genetics* **20**, 43–55 (2014).

17. Berry, D. L. & Baehrecke, E. H. Growth arrest and autophagy are required for salivary gland cell degradation in Drosophila. *Cell* **131**, 1137–1148 (2007).

18. Mondragon, A. A. *et al.* Lysosomal Machinery Drives Extracellular Acidification to Direct Non-apoptotic Cell Death. *Cell Rep.* **27**, 11-19.e3 (2019).

19. Kinet, M. J. *et al.* HSF-1 activates the ubiquitin proteasome system to promote non-apoptotic developmental cell death in C. elegans. *Elife* **5**, e12821 (2016).

20. Abraham, M. C., Lu, Y. & Shaham, S. A morphologically conserved nonapoptotic program promotes linker cell death in Caenorhabditis elegans. *Dev.Cell* **12**, 73–86 (2007).

21. Lamkanfi, M. & Dixit, V. M. Mechanisms and functions of inflammasomes. *Cell* **157**, 1013–1022 (2014).

22. Broz, P., Pelegrín, P. & Shao, F. The gasdermins, a protein family executing cell death and inflammation. *Nat. Rev. Immunol.* **20**, 143–157 (2020).

23. Aram, L., Yacobi-Sharon, K. & Arama, E. CDPs: Caspase-dependent non-lethal cellular processes. *Cell Death Differ.* **24**, 1307–1310 (2017).

24. Baena-Lopez, L. A. All about the caspase-dependent functions without cell death. *Semin. Cell Dev. Biol.* **82**, 77–78 (2018).

25. Arama, E., Baena-Lopez, L. A. & Fearnhead, H. O. Non-lethal message from the Holy Land: The first international conference on nonapoptotic roles of apoptotic proteins. *FEBS J.* (2020) doi:10.1111/febs.15547.

26. Gorelick-Ashkenazi, A. *et al.* Caspases maintain tissue integrity by an apoptosis-independent inhibition of cell migration and invasion. *Nat. Commun.* **9**, 2806 (2018).

27. Feinstein-Rotkopf, Y. & Arama, E. Can't live without them, can live with them: Roles of caspases during vital cellular processes. *Apoptosis* **14**, 980–995 (2009).
